# Hepatitis B Awareness and Knowledge in Asian Communities in British Columbia

**DOI:** 10.1155/2016/4278724

**Published:** 2016-03-29

**Authors:** Alan Hoi Lun Yau, Jo-Ann Ford, Peter Wing Cheung Kwan, Jessica Chan, Queenie Choo, Tim K. Lee, Willie Kwong, Alan Huang, Eric Yoshida

**Affiliations:** ^1^Division of Gastroenterology, Department of Medicine, University of British Columbia, GLDHCC, 2775 Laurel Street, 5th Floor, Vancouver, BC, Canada V5Z 1M9; ^2^S.U.C.C.E.S.S., 555 Carrall Street, Vancouver, BC, Canada V6B 2J8; ^3^Cancer Control Research Program, BC Cancer Agency, 675 West 10th Avenue, Vancouver, BC, Canada V5Z 1L3; ^4^School of Population and Public Health, University of British Columbia, 2206 East Mall, Vancouver, BC, Canada V6T 1Z9

## Abstract

*Background.* Our study examined hepatitis B virus (HBV) awareness and knowledge in Asian communities in British Columbia (BC).* Methods.* A statistical random sample representation of Chinese, Korean, Filipino, South Asian, and Southeast Asian populations in Greater Vancouver was surveyed by telephone. Multiple logistic regression analysis was performed to identify predictors of HBV knowledge.* Results.* General awareness of HBV was reported in 78.8% (798/1013). HBV awareness was the highest in Chinese (89%) and Filipino (88%) populations and the lowest in the South Asian (56%) population. “Reasonable” knowledge of HBV was elicited in 76.8% (778/1013). Higher HBV knowledge was associated with younger age (*p* = 0.014), higher education (*p* < 0.0001), Chinese ethnicity (*p* < 0.0001), and use of media (*p* = 0.01) and Internet (*p* = 0.024) for health information. Compared to the Chinese (OR = 1.0) population, “reasonable” knowledge of HBV was lower in Korean (OR = 0.3, 95% CI: 0.1–0.5), Filipino (OR = 0.3, 95% CI: 0.2–0.6), South Asian (OR = 0.3, 95% CI: 0.2–0.4), and Southeast Asian (OR = 0.3, 95% CI: 0.1–0.6) populations. 54.8% (555/1013) felt that HBV education was inadequate and 80.1% (811/1013) preferred HBV education in their native languages.* Conclusion.* Compared to the Chinese population, other Asian communities in BC have lower HBV awareness and knowledge. Public education should target older and less educated and Korean, Filipino, South Asian, and Southeast Asian populations in their native languages via media and Internet.

## 1. Introduction

In Asia, hepatitis B virus (HBV) infection is highly endemic where 70–90% of the population become infected by the age of 40 and where 8–20% are chronic carriers [1]. Canada is a country with low endemicity for HBV where 0.8% of the population (180,000 individuals) are chronic carriers [2]. However, among the Canadian provinces, British Columbia (BC) has the highest proportion of Asians in its population, which includes Chinese (10.6%), South Asian (6.5%), East and Southeast Asian (16.0%), and East Indian (5.7%) populations [3]. In BC, 6% of the Chinese immigrants are chronic carriers of HBV [4]. Chronic HBV infection results in cirrhosis in 20–40% [5], increases the risk of hepatocellular carcinoma 100-fold [5], and leads to 500,000–700,000 deaths annually worldwide [6].

Given the significant morbidity and mortality associated with chronic HBV infection, the previously reported rates of HBV vaccination (29–38%) and screening (39–57%) among Chinese immigrants in BC are suboptimal and concerning [4, 7]. An association between increased rates of vaccination and screening for HBV and a greater knowledge of HBV has been consistently demonstrated in multiple studies [7–16]. Hence, continuing efforts in community education may promote uptake of preventive measures and therapeutic interventions for HBV, thereby alleviating the increasing burden of HBV on the Canadian health care system. However, in a survey conducted in BC in 2005, only 13% of the Chinese immigrants felt that HBV education was adequate in the community [17]. In order to improve the effectiveness of community health education campaigns, it is essential to gain a thorough understanding of the public awareness and knowledge of HBV.

We performed a telephone survey on a statistical random sample representation of the Asian populations residing within the Greater Vancouver Regional District (GVRD). Our aims were to (1) evaluate public awareness and knowledge of HBV; (2) identify sociodemographic variables associated with HBV knowledge; and (3) gather public opinion on the preferred source and language of HBV education in the community.

## 2. Methods

### 2.1. Population Sampling

From April 16, 2012, to May 4, 2012, 1013 individuals participated in a telephone interview conducted in one of seven different languages by an external company called Select Field Services. Prospective respondents were called at least three times at different times of the day before a prospective respondent was dropped and replaced with another. A sampling frame based on pertinent surnames of all targeted ethnic households residing within GVRD was purchased to obtain a list of telephone numbers which were imported into a Computer-Assisted Telephone Interviewing (CATI) system. A total sample size of 1000 to 1200 successful interviews was predetermined in order to achieve the statistical representation and to produce survey results with a 95% confidence interval for the outcome of HBV awareness and knowledge. Sampling quotas are preset in proportion to the overall size of each ethnic group via proportional random sampling. Random selection of households and of a particular adult within each selected household was performed by CATI. The ethnic groups sampled were Chinese (Mainland China, Hong Kong, and Taiwan); Korean; Filipino; South Asian (East Indian, Bangladeshi, Bengali, Goan, Gujarati, Kashmiri, Nepali, Pakistani, Punjabi, Sinhalese, Sri Lankan, and Tamil); and Southeast Asian (Vietnamese, Laotian, Cambodian, Singaporean, Malaysian, Indonesian, and Burmese) populations. The study was approved by the University of British Columbia Behavioural Research Ethics Board.

### 2.2. Survey Instrument

A standardized questionnaire was developed in English, translated into five different languages, administered in the respondents' native language, and back-translated to English to ensure accuracy. The questionnaire contained five sections including questions on sociodemographics, health care utilization, HBV awareness, HBV knowledge, and HBV education (see Supplementary Material available online at http://dx.doi.org/10.1155/2016/4278724).

### 2.3. Statistical Analysis

HBV knowledge was considered “reasonable” if respondents correctly answered 7 or more of the 13 questions. Associations between demographic and knowledge variables were assessed using *χ* ^2^ test. Independent predictors of HBV knowledge were identified by multiple logistic regression forward stepwise analysis. Tests for trend across category groupings of age and education were conducted by treating the participant age and education level as continuous variables. Statistical significance was achieved if two-tailed *p* < 0.05. Data were analyzed using SPSS version 20.0 (IBM Corporation, USA) for Windows XP (Microsoft Corporation, USA).

## 3. Results

### 3.1. Sample Characteristics (Table 1)

A total of 1013 individuals participated in the survey. The majority (66%) of respondents were below 55 years of age. There were similar numbers of male and female respondents (44% and 56%, resp.). The ethnicities of the respondents were Chinese (51.2%), Korean (6.2%), Filipino (11.6%), South Asian (27.0%), and Southeast Asian (3.8%). The majority of respondents were relatively well educated with postsecondary education in 20% and university education in 45%. The majority were bilingual (88%) and had family physicians (93%).

### 3.2. HBV Testing, Diagnosis, and Treatment

HBV testing was performed in 48.3% (489/1013), and HBV was diagnosed in 5.6% (57/1013), of which 70.2% (40/57) were not receiving treatment for HBV, and 87.7% (50/57) were not taking medication for HBV. A family history of HBV infection was present in 8%.

### 3.3. HBV Awareness (Table 2)

General awareness of HBV was reported in 78.8% (798/1013). HBV awareness was the highest in the Chinese (89%) and Filipino (88%) populations and the lowest in the South Asian (56%) population. 48.9% (495/1013) felt that HBV was a concern for the community.

### 3.4. HBV Knowledge (Table 3)

76.8% (778/1013) demonstrated a “reasonable” level of HBV knowledge. A low proportion of individuals were aware that HBV could lead to chronic disease (61%), cirrhosis (62%), and hepatocellular carcinoma (62%). Perinatal and sexual transmission of HBV were recognized by 68% and 37% of respondents, respectively; however, only 26% of respondents knew that HBV cannot be transmitted by sharing food or utensils with an infected person. The majority of respondents knew that HBV can be diagnosed by blood tests (84%) and that HBV can be prevented by vaccine (71%), but only 53% were aware of the effective treatment for HBV.

### 3.5. Multivariate Analysis (Tables 4 and 5)

Higher HBV knowledge was statistically significantly associated with younger age (*p* = 0.014), higher education (*p* < 0.0001), Chinese ethnicity (*p* < 0.0001), and use of media (*p* = 0.001) and Internet (*p* = 0.024) for health information. Knowledge that HBV may cause cirrhosis was statistically significantly associated with higher education (*p* < 0.0001), Chinese ethnicity (*p* < 0.0001), and use of media for health information (*p* = 0.009). Knowledge that HBV may cause liver cancer was statistically significantly associated with Chinese ethnicity (*p* < 0.0001) (data not shown). Compared to the Chinese (OR = 1.0) population, “reasonable” knowledge of HBV was lower in Korean (OR = 0.3, 95% CI: 0.1–0.5), Filipino (OR = 0.3, 95% CI: 0.2–0.6), South Asian (OR = 0.3, 95% CI: 0.2–0.4), and Southeast Asian (OR = 0.3, 95% CI: 0.1–0.6) populations.

### 3.6. HBV Education (Table 6)

54.8% (555/1013) felt that HBV education was inadequate in the community. 80.1% (811/1013) preferred HBV education in their native languages. The main sources of health information were doctor's office (58%), Internet (41%), and media (40%). There was a significant association (*p* < 0.0001) between younger age and use of Internet for health information (56.4% at the age of 19–24, 55.2% at the age of 25–39, 39.9% at the age of 40–54, and 28.0% at the age of ≥55). Only 20% of respondents felt that HBV has received sufficient funding support from the BC government for preventive health education and patient care, compared to tuberculosis (23%), chronic obstructive pulmonary disease (24%), hypertension (34%), HIV/AIDS (34%), diabetes (41%), and cancer (42%).

## 4. Discussion

In the present study, HBV awareness was reported in 79% of the respondents, which is consistent with the rate of 68–85% in previous studies examining Chinese immigrants in BC [7, 17]. However, awareness may not reflect knowledge, and many respondents in our study had misconceptions in the natural history, transmission modes, and health consequences of HBV. Furthermore, although 84% of respondents knew that HBV can be diagnosed by blood tests, only 48% have ever been tested for HBV, despite having a family physician in 93%. This latter finding suggests knowledge deficits of family physicians in the availability of effective treatment for HBV, further reinforcing the need for greater educational efforts in HBV among primary care physicians.

Chinese ethnicity was identified as a positive predictor of higher HBV knowledge in our study. Significantly more Chinese respondents knew that HBV may cause cirrhosis or liver cancer, and overall HBV knowledge was higher in the Chinese population compared to other Asian ethnic groups. Previous studies have also reported knowledge deficits in HBV in Korean [18, 19], South Asian [20–24], and Southeast Asian [10, 13, 15, 16, 25–31] populations. The differences in HBV knowledge among Asian populations in BC may be attributed to the introduction of HBV educational campaigns to the local Chinese media via radio and newspaper in 2007. Indeed, the use of media for health information was identified as a positive predictor of higher HBV knowledge in our study. Hence, our findings indicate an urgent need to expand community education on HBV to other Asian ethnic groups.

Younger age was found to be a positive predictor of higher HBV knowledge in our study, in contrast to a previous study performed in 2005 which identified an association with older age among Chinese immigrants in BC [17]. A possible explanation is that the Internet is being utilized more frequently in recent years by younger individuals for health information and this was confirmed by the significant association between younger age and use of Internet for health information in our study. A study in Taiwan evaluating an education program via the Internet for university students found that it was welcomed by 68% and that it significantly improved their HBV knowledge [32]. Indeed, the use of Internet for health information was identified as a positive predictor of higher HBV knowledge in our study.

Higher education was another positive predictor of higher HBV knowledge in our study. Such an association has been well documented in the literature [7, 12, 17–21, 27, 33–39]. In addition, both higher education [7, 14, 40, 41] and higher HBV knowledge [7–16] have been associated with increased rates of HBV screening and vaccination. On the other hand, greater levels of HBV stigma have been associated with a decreased likelihood of HBV screening [8]. These findings provide support for the notion that knowledge dictates behavior in the setting of HBV.

Respondents in our study expressed high levels of concern (49%) and desire (55%) for further HBV education in the community. Moreover, many respondents felt that, compared to other major chronic diseases, HBV is receiving the least funding support from the BC government for preventive health education. The most common source of health information reported by our respondents was doctor's office which has been shown to be effective in increasing HBV knowledge and screening. Indeed, student-run clinics at the University of California San Francisco providing community health education were shown to significantly improve the HBV knowledge of patients [42]. In addition, physician recommendation was identified as a positive predictor of HBV testing in Vietnamese Americans [15]. Finally, 80% of our respondents indicated a preference for HBV education in their native languages, even though 88% were bilingual. Indeed, language difficulty was identified as a significant barrier to health care in HBV among Chinese immigrants in Toronto [5]. On the other hand, English as a second language (ESL) educational curriculum was found to be effective in increasing HBV knowledge among Chinese immigrants in Vancouver [43]. These findings highlight the importance of providing community health education in a culturally sensitive manner.

There were several limitations in our study. First, households with unlisted telephone numbers were not included in our sample. Second, respondents to the survey may have had higher levels of knowledge and increased rates of testing compared to those who refused to participate. Third, self-reported rates of HBV screening are subject to recall bias and may be inaccurate. Finally, our study did not collect data on rates of HBV vaccination.

In summary, there is an urgent need to raise public awareness and knowledge of HBV in order to avert its perpetuation in the community. HBV education should discuss (1) screening of high-risk individuals from highly endemic areas; (2) routes of transmission and precautions to avoid infecting others; (3) vaccination of family members and sexual contacts; (4) monitoring of ALT and HBV DNA and surveillance for HCC with abdominal ultrasound; and (5) potential benefit of highly efficacious and well tolerated antiviral therapy in chronic carriers.

## 5. Conclusion

Compared to the Chinese population, other Asian ethnic groups appear to have less awareness and knowledge of HBV. There are both a concern and a desire for further HBV education among Asian immigrant populations in BC. Public education should target older and less educated and South Asian, Filipino, Korean, and Southeast Asian populations in their native languages via media and Internet.

## Supplementary Material

A standardized questionnaire was developed in English, translated into five different languages, administered in the respondents' native language, and back-translated to English to ensure accuracy. The questionnaire contained five sections including questions on sociodemographics, health care utili-zation, HBV awareness, HBV knowledge, and HBV education.

## Figures and Tables

**Table 1:** Baseline characteristics of survey respondents.

Characteristics	*N* (%)
Age	
19–24	78 (7.7)
25–39	252 (24.9)
40–54	336 (33.2)
≥55	343 (33.9)
Gender	
Male	448 (44.2)
Female	565 (55.8)
Ethnicity	
Chinese	519 (51.2%)
South Asian	274 (27.0)
Filipino	118 (11.6)
Korean	63 (6.2)
Southeast Asian	39 (3.8)
Status in Canada	
Citizen	751 (74.1)
Permanent resident	239 (23.6)
Nonresident (temporary foreign worker, student, or visitor)	23 (2.3)
Language spoken at home	
Chinese (Cantonese and Mandarin)	513 (50.6)
English	83 (8.2)
Korean	48 (4.7)
Southeast Asian languages	104 (10.3)
South Asian languages	256 (25.3)
Other languages	9 (0.9)
Second language at home	
English	807 (79.7)
French	3 (0.3)
Spanish	4 (0.4)
None	126 (12.4)
Education	
Primary/elementary	91 (9.0)
Secondary	253 (25.0)
Postsecondary	199 (19.6)
University	453 (44.7)
Working	
Yes	484 (47.8)
No	525 (51.8)
Having a family doctor	
Yes	944 (93.2)
No	68 (6.8)
Physical checkup	
Yes	729 (72.0)
No	284 (28.0)

**Table 2:** HBV awareness of survey respondents.

Hepatitis B awareness *N* (%)	Total (*N* = 1013)	Chinese (*N* = 522)	South Asian (*N* = 274)	Filipino (*N* = 117)	Korean (*N* = 60)	Southeast Asian (*N* = 39)
Are you aware of a disease called hepatitis B?						
Yes	798 (78.8)	465 (89.0)	152 (55.5)	103 (88.1)	46 (76.2)	32 (82.1)
No	184 (18.2)	49 (9.4)	109 (39.8)	10 (8.5)	10 (17.5)	5 (12.8)
Uncertain	31 (3.1)	8 (1.5)	13 (4.7)	4 (3.4)	4 (6.3)	2 (5.1)
Is hepatitis B a concern of the Asian community?						
Yes	495 (48.9)	297 (57.0)	90 (32.8)	56 (48.3)	32 (54.0)	18 (46.2)
No	229 (22.6)	89 (17.0)	81 (29.6)	29 (24.6)	20 (33.3)	10 (25.6)
Uncertain	289 (28.5)	136 (26.0)	103 (37.6)	32 (27.1)	8 (12.7)	11 (28.2)

**Table 3:** HBV knowledge of survey respondents.

Hepatitis B knowledge	*N* (%)
Knowledge level	
Reasonable knowledge (≥7 correct answers)	778 (76.8)
Not reasonable knowledge (≤6 correct answers)	235 (23.2)
Low knowledge (≤4 correct answers)	130 (12.8)
Knowledge question [correct response]	
In most cases, hepatitis B is only a temporary infection (like the flu) [false]	621 (61.3)
Hepatitis B is a cause of cirrhosis (severe scarring of liver) [true]	625 (61.7)
Hepatitis B is a cause of liver cancer [true]	631 (62.3)
Hepatitis B can be transmitted through an infected mother to her child [true]	689 (68.0)
Hepatitis B is a sexually transmitted disease [true]	379 (37.4)
Hepatitis B can be transmitted by sharing food or utensils of an infected person [false]	267 (26.4)
Hepatitis B can be diagnosed by blood tests [true]	854 (84.3)
A vaccine that can prevent hepatitis B exists [true]	720 (71.1)
There is effective treatment for hepatitis B [true]	538 (53.1)
Hepatitis B is more common in China and Asia versus North America [true]	619 (61.1)
Little children can have hepatitis B [true]	760 (75.0)
Hepatitis B affects adults [true]	669 (66.0)
Hepatitis B is preventable [true]	800 (79.0)

**Table 4:** Predictors of HBV knowledge from multivariate analysis.

Characteristics	Odds ratio (95% CI)	*p* value
Age		
19–24	1.8 (0.9–3.5)	0.084
25–39	1.7 (1.1–2.7)	0.022^*∗*^
40–54	2.0 (1.3–3.0)	0.001^*∗*^
≥55	1.0	—
		*p* (trend) = 0.014^*∗*^
Education		
Primary/elementary	0.3 (0.1–0.5)	<0.0001^*∗*^
Secondary	0.5 (0.3–0.7)	0.001^*∗*^
Postsecondary	0.7 (0.4–1.1)	0.116
University or above	1.0	—
		*p* (trend) < 0.0001^*∗*^
Ethnicity		
Chinese (Cantonese and Mandarin)	1.0	—
South Asian	0.3 (0.2–0.4)	<0.0001^*∗*^
Filipino	0.3 (0.2–0.6)	<0.0001^*∗*^
Korean	0.3 (0.1–0.5)	<0.0001^*∗*^
Southeast Asian	0.3 (0.1–0.6)	0.001^*∗*^
Media for health information		
No	0.6 (0.4–0.8)	0.001^*∗*^
Yes	1.0	—
Internet for health information		
No	0.6 (0.4–0.9)	0.024^*∗*^
Yes	1.0	—

^*∗*^Statistically significant at *p* < 0.05.

**Table 5:** Predictors of knowledge that HBV may cause cirrhosis from multivariate analysis.

Characteristics	Odds ratio (95% CI)	*p* value
Education		
Primary/elementary	0.3 (0.2–0.5)	<0.0001^*∗*^
Secondary	0.7 (0.5–1.0)	0.073
Postsecondary	0.7 (0.5–1.0)	0.039^*∗*^
University or above	1.0	—
		*p* (trend) < 0.0001^*∗*^
Ethnicity		
Chinese (Cantonese and Mandarin)	1.0	—
South Asian	0.2 (0.2–0.3)	<0.0001^*∗*^
Filipino	0.3 (0.2–0.5)	<0.0001^*∗*^
Korean	0.4 (0.2–0.8)	<0.0001^*∗*^
Southeast Asian	0.1 (0.1–0.3)	0.001
Media for health information		
No	0.7 (0.5–0.9)	0.009^*∗*^
Yes	1.0	—
Doctor's office for health information		
No	1.6 (1.2–2.2)	0.001^*∗*^
Yes	1.0	—

^*∗*^Statistically significant at *p* < 0.05.

**Table 6:** Opinions on HBV education by survey respondents.

HBV education	Total (*N* = 1013)	Chinese (*N* = 522)	South Asian (*N* = 274)	Filipino (*N* = 117)	Korean (*N* = 60)	Southeast Asian (*N* = 39)
Is hepatitis B education adequate in the community?						
Yes	208 (20.5)	66 (12.7)	62 (22.3)	42 (35.6)	30 (49.2)	8 (20.5)
No	555 (54.8)	322 (61.7)	153 (55.8)	40 (33.9)	20 (33.3)	21 (53.8)
Uncertain	250 (24.7)	134 (25.6)	60 (21.9)	35 (30.5)	10 (17.5)	10 (25.6)
Which language is more effective for hepatitis education in the Asian community?						
Native language	811 (80.1)	446 (85.5)	244 (89.1)	48 (40.7)	47 (76.2)	27 (69.2)
English	51 (5.1)	15 (2.9)	11 (4.0)	15 (12.7)	6 (9.5)	5 (12.8)
No difference	152 (14.8)	61 (11.6)	19 (6.9)	54 (46.6)	8 (14.3)	7 (17.9)
What is your main source of health information?						
Doctor's office	588 (58.1)	228 (43.7)	202 (73.7)	88 (75.4)	40 (65.1)	30 (76.9)
Pharmacist	182 (17.9)	73 (13.9)	24 (8.8)	37 (31.4)	32 (52.4)	15 (38.5)
Family and friends	274 (27.5)	124 (23.7)	47 (17.2)	55 (46.6)	33 (54.0)	20 (51.3)
Internet	436 (40.9)	249 (47.6)	61 (22.3)	71 (61.0)	16 (25.4)	18 (46.2)
Media	395 (40.4)	243 (46.6)	75 (27.4)	58 (49.2)	14 (22.2)	20 (51.3)
School	132 (13.4)	79 (15.2)	5 (1.8)	34 (28.8)	10 (15.9)	8 (20.5)
Other	61 (6.0)	30 (5.8)	17 (6.2)	12 (10.2)	1 (1.6)	1 (2.6)
Uncertain	20 (1.7)	11 (2.1)	5 (1.8)	0	0	1 (2.6)
Is hepatitis B education receiving sufficient governmental funding?						
Sufficient	190 (19.0)	78 (14.9)	51 (18.8)	34 (31.2)	16 (26.7)	11 (29.7)
Insufficient	290 (29.0)	209 (40.0)	43 (15.9)	17 (15.6)	12 (20.0)	9 (24.3)
Neither	90 (9.0)	37 (7.1)	5 (1.8)	26 (23.9)	13 (21.7)	7 (18.9)
Uncertain	430 (43.0)	199 (38.0)	171 (63.1)	33 (30.3)	19 (31.7)	10 (27.0)
