# Supplementary material for: Hepatitis B Awareness and Knowledge in Asian Communities in British Columbia
Source: Can J Gastroenterol Hepatol. 2016 Mar 29;2016:4278724. doi: 10.1155/2016/4278724 (PMC4904637; doi:10.1155/2016/4278724)
Supplement: Supplementary file 1 — A standardized questionnaire was developed in English, translated into five different languages, administered in the respondents' native language, and back-translated to English to ensure accuracy. The questionnaire contained five sections including questions on sociodemographics, health care utili-zation, HBV awareness, HBV knowledge, and HBV education. [file 4278724.f1.pdf]

Prepared by:
S.U.C.C.E.S.S.

HEPATITIS B AWARENESS SURVEY IN ASIAN COMMUNITIES

March 26, 2012

Good morning / afternoon / evening. My name is __________. I am calling from Select Field Services. We are conducting a community health survey on behalf of S.U.C.C.E.S.S., UBC Medical School, Division of Gastroenterology, and the BC Hepatitis Program at Vancouver General Hospital. We are speaking with **BC residents of the ethnic communities** to answer a few questions regarding their opinion and understanding of the Hepatitis B disease. The survey will take less than 15 minutes.

S1 Are you a BC resident 19 years of age or older?

Yes - SPEAKING, CONTINUE	1=> MONIT
No	2=> /S2
Refused – LOG **(THANK AND TERMINATE)**	99=> /INT

S2. Can I speak to anyone else who lives at this residence that is 19 years of age or older and normally resides in BC?

Yes - **REPEAT INTRODUCTION WITH NEW RESPONDENT & CONTINUE**	1=> HELLO
No eligible respondent resides in household. **THANK & TERMINATE** – **LOG**	2=> /INT
No, eligible respondent is not available - **ARRANGE CALLBACK**	3=> /INT
Don't Know/Refused – LOG **(THANK AND TERMINATE)**	99=> /INT

MONIT This call may be monitored or audio taped for quality control and evaluation purposes.

Confidentiality and Respondent Rights I will assure you of the confidentiality and personal anonymity of the interview. Your responses in the in the survey will be protected. Only researcher of this survey will have access to the information and analyze it on a group basis. You have the right to terminate the interview at any time or not to answer any question if they are not comfortable to provide an answer. You have to right to speak to a call centre supervisor if you are not satisfied with the calling interviewer. Can I have your consent to continue?

PRESS TO CONTINUE 1

A. Personal Information

1. What is your ethnic origin?
[DO NOT READ LIST]

Chinese	1
Korean	2
Filipino	3
Vietnamese	4
Cambodian	5
Laotian	6
Bangladeshi	7
Bengali	8
Goan	9
Gujarati	10
Kashmiri	11
Nepali	12
Pakistani	13
Punjabi	14
Sinhalese	15

Sri Lankan	16	
Tamil	17	
East Indian	18	
Other **(THANK AND TERMINATE)**	88	=> /INT
Don't Know/Refused **(THANK AND TERMINATE)**	99	=> /INT

GENDER ******* DON'T READ - RECORD GENDER BY OBSERVATION *******

Male	1
Female	2

2. Which of the following age groups do you fall into?

19-25	1
25-39	2
40-54	3
55 years or older	4
Refused **(DO NOT READ)**	99

3. What is your status in Canada?

Citizen	1
Permanent resident	2
Temporary foreign worker	3
Visitor	4
Student	5
Other [Please specify] **(DO NOT READ)**	88
Don't know **(DO NOT READ)**	98
Refused **(DO NOT READ)**	99

4. How long have you lived in Canada?

Year(s) _____
Month(s) _____

5. Which city do you live in Metro Vancouver?

6. Where is your birth place?

Hong Kong	1
Mainland China	2
Taiwan	3
Vietnam	4
Cambodia	5
Laos	6
Korea	7
Philippines	8
India	9
Bangladesh	10
Bengal	11
Goa	12
Gujar	13
Kashmir	14
Nepal	15
Pakistan	16
Punjab	17
Sri Lanka	19
Other Asian countries [Please specify]	88
Don't know **(DO NOT READ)**	98
Refused **(DO NOT READ)**	99

7. What is your main language spoken at home?
[One answer only]

Cantonese	1
Mandarin	2
English	3
Korean	4
Tagalog	5
Hindi	6
Urdu	7
Punjabi	8
Bengali	9
Gujarati	10
Tamil	11
Sinhalese	12
Nepali	13
Other language [Please specify]	88
Refused **(DO NOT READ)**	99

8. What second language(s) do you speak?
[Multiple answers allowed]

English	1
French	2
Spanish	3
None	4
Other [Please specify]	88
Refused **(DO NOT READ)**	99

9. What is the highest education level you have completed?

Primary/elementary school	1
Secondary school	2
Post-secondary college	3
University or above	4
Refused **(DO NOT READ)**	99

10. Are you currently employed?

Working	1
Not working	2
Refused **(DO NOT READ)**	99

11. Do you have a family doctor?

Yes	1
No	2
Don't know **(DO NOT READ)**	98
Refused **(DO NOT READ)**	99

12. Do you have your physical check-up, including a blood test, done annually or regularly by a family/medical doctor?

Yes	1
No	2
Refused **(DO NOT READ)**	99

B. Hepatitis B Awareness Questions

For the following questions please answer **Yes**, **No**, or **Uncertain**. **[READ LIST]**

	Yes 1	No 2	Uncertain 98
13. Are you aware of a disease called hepatitis B?			
14. In your opinion, is hepatitis B a concern of the Asian community?			
15. In your opinion, is hepatitis B education adequate in the community?			
16. Have you ever been tested for hepatitis B?			
17. Have you been diagnosed with having hepatitis B?			
18. (if yes to 17) Are you currently being treated by your doctor for hepatitis B?			
19. (if yes to 17)Are you currently taking medication for hepatitis B?			
20. Has anyone in your family been diagnosed of having hepatitis B?			

21. Are you familiar with a medical condition called cirrhosis (severe scarring of liver)?

C. Hepatitis B Knowledge Test Questions

Please tell me if the following statements are **True or False**. **[READ LIST]**

	True 1	False 2	Don't Know 98
22. In my opinion, in most cases hepatitis B is only a temporary infection (like the flu)			
23. In my opinion, small children can have hepatitis B.			
24. In my opinion, hepatitis B is a cause of liver cancer.			
25. In my opinion, hepatitis B is more common in China and Asia vs. North America.			
26. In my opinion, hepatitis B affects adults.			
27. In my opinion, there is effective treatment for hepatitis B.			
28. In my opinion, hepatitis B is a cause of cirrhosis (severe scarring of liver)			
29. In my opinion, hepatitis B is preventable.			
30. In my opinion, hepatitis B is a sexually transmitted disease.			
31. A vaccine that can prevent hepatitis B exits.			
32. In my opinion, hepatitis B can be transmitted through an infected mother to her child.			
33. In my opinion, hepatitis B can be diagnosed by blood tests.			
34. In my opinion, hepatitis B can be transmitted by sharing food or utensils of an infected person.			

D. Source of Medical Information Questions

35. From what source do you usually obtain information for medical or health needs? **[Multiple answers allowed]**

Doctor's office	1
Pharmacist	2
Family & friends	3
School	4
Media	5
Internet	6
Other **[please specify]**	88
Don't know **(DO NOT READ)**	98
Refused **(DO NOT READ)**	99

36. Are you familiar of the BC Hepatitis Program?

Yes	1
No	2
Refused **(DO NOT READ)**	99

37. In your opinion, which would be more effective: if hepatitis education is provided in ethnic languages or in English for the Asian community in Metro Vancouver? Or it would make no difference?

In ethnic languages	1
English	2
No Difference	3

E. BC Government & Political Support Questions

In your opinion, the extent to which the following chronic diseases have received sufficient or insufficient funding support by the BC Government for preventive health education and patient care?

	Very insufficient				Very sufficient	Don't Know
	1	2	3	4	5	98
**[Randomize]**						
38. Heart disease (hypertension)						
39. Cancer						
40. Diabetes						
41. TB (Tuberculosis)						
42. COPD (Chronic obstructive pulmonary disease - a long-term lung disease often caused by smoking)						
43. Hepatitis B						
44. HIV /AIDS (Acquired Immune Deficiency Syndrome)						

Thank you!
